# Ogilvie's Syndrome following Cardioversion for Atrial Fibrillation

**DOI:** 10.1155/2014/841491

**Published:** 2014-08-19

**Authors:** Moh'd Al-Halawani, Juanito Savaille, Mohammad Thawabi, Yazan Abdeen, Richard A. Miller, Andre A. Fedida

**Affiliations:** Department of Internal Medicine, Saint Michael's Medical Center, Seton Hall University School of Health and Medical Sciences, Newark, NJ 07102, USA

## Abstract

Acute colonic pseudoobstruction, also known as Ogilvie's syndrome, is characterized by distension of the colon in the absence of a mechanical obstruction as evident by abdominal radiography. This syndrome is usually treated conservatively; however, medical or surgical therapies can be employed in refractory cases. Ogilvie's syndrome has been reported following cardiac events, such as myocardial infarction, heart failure, and cardiac bypass surgeries. We report the first case of Ogilvie's syndrome following synchronized electric cardioversion for atrial fibrillation.

## 1. Introduction

Acute colonic pseudoobstruction (ACPO), also known as Ogilvie's syndrome, is characterized by distension of the colon in the absence of a mechanical obstruction. The most severe complications are perforation and ischemia of the colon [1]. Hospitalized patients are at risk of developing Ogilvie's syndrome because of underlying medical or surgical conditions. Predisposing factors associated with the syndrome include metabolic disturbances, cardiac disease, infections, organ failure, trauma, malignancy, drugs, surgeries, and autoimmune and neurological disorders [2]. We are reporting a case of acute colonic pseudoobstruction that developed in a patient presenting with atrial fibrillation after undergoing cardioversion.

## 2. Case Presentation

**Figure 1:**
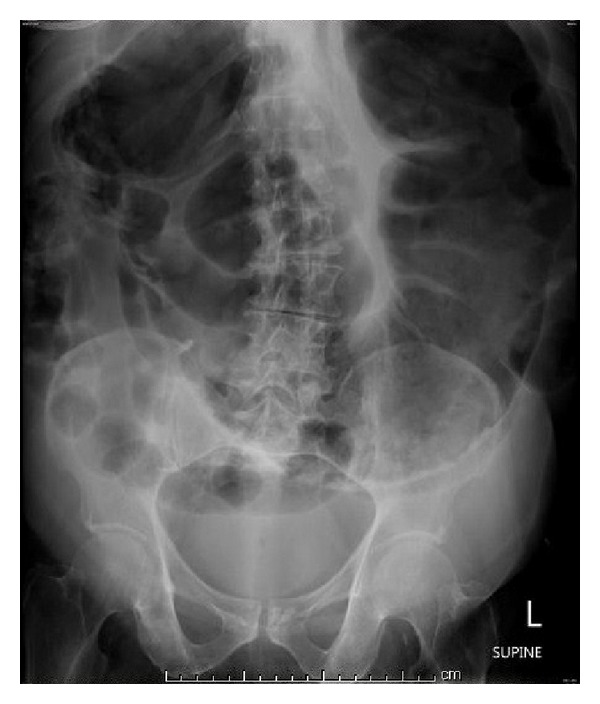
A plain abdominal X-ray showing the colonic dilatation with a cutoff at the rectum.

**Figure 2:**
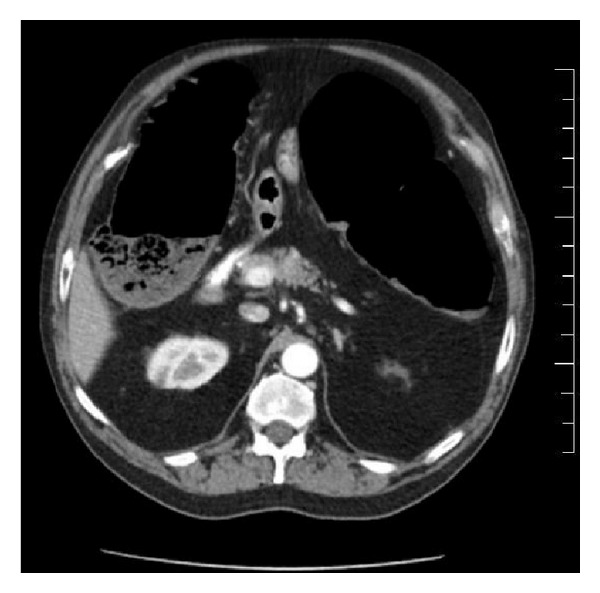
Axial CT scan of the abdomen showing the dilated large bowel.

We report a case of a 68-year-old male with a past medical history of chronic obstructive pulmonary disease and hypertension admitted for shortness of breath and palpitations of two-day duration. The shortness of breath was associated with productive cough of clear sputum. Initial vital signs were blood pressure 153/94 mmHg, heart rate of 127 beats per minute, and respiratory rate of 32 breaths per minute and oxygen saturation was 95% on room air. Physical examination was significant for irregularly irregular pulse, normal heart sounds, diffuse rhonchi and wheezing bilaterally, and a soft, nontender abdomen with active bowel sounds. Initial lab results were within normal limits. Electrocardiogram revealed atrial fibrillation with rapid ventricular response. The patient was started on IV Diltiazem and anticoagulated with heparin and warfarin. Two days into his admission the patient underwent a transesophageal echocardiogram which did not show a clot in the left atrial appendage. Afterwards, he had synchronized cardioversion at 120 Joules, with a successful return to normal sinus rhythm, and was started on flecainide. Two days after cardioversion, the patient started complaining of constipation, abdominal pain, and abdominal distension. Lab results were all within normal limits. The patient has no past-surgical history. A flat plate abdominal X-ray showed large bowel dilatation (Figure 1). Subsequently, a CT scan of the abdomen with oral contrast was ordered which revealed a diffusely dilated colon—mainly the ascending and transverse colon—with the largest diameter being 11 cm and no physical obstruction (Figure 2). The patient was diagnosed with acute colonic pseudoobstruction (Ogilvie's syndrome). Conservative management was started by giving the patient nothing by mouth and intravenous fluids. Nasogastric tube to intermittent suction and a rectal tube were placed with no improvement in the patient's symptoms. The patient was then given two doses of neostigmine 1 mg intravenously in two consecutive days with a moderate, nonsustained response. He underwent colonoscopic decompression the following day, which further revealed dark dusky mucosa in the right colon suggestive of ischemia. Despite aggressive medical management and colonic decompression the patient had recurrence of distension of his abdomen and underwent exploratory laparotomy and placement of cecostomy tube. The patient clinically improved and was transferred to a subacute rehabilitation facility. Three weeks later, the patient had removal of the cecostomy tube without any further complications.

## 3. Discussion

Ogilvie's syndrome is a rare disorder characterized by a massive colonic dilatation in the absence of mechanical obstruction [1]. It was first described by Ogilvie in 1948 who described two patients with colonic ileus resulting from malignant infiltration of the celiac plexus [3].

Vanek and Al-Salti reported the conditions associated with Ogilvie's syndrome in 400 patients. It was associated with infectious diseases, nonoperative trauma, myocardial infarction, heart failure, obstetric and gynecologic surgeries, abdominal-pelvic surgery, neurological diseases (such as Parkinson's disease, spinal cord injury, multiple sclerosis, or Alzheimer's disease), orthopedic surgery (knee and hip replacements, scoliosis surgery), and other miscellaneous medical conditions (metabolic, cancer, respiratory failure, and renal failure) [4]. It has also been reported with other major operations, such as renal transplantation and cardiac bypass surgery, as well as transcatheter arterial chemoembolization therapy and decompensated ischemic cardiomyopathy [5–7].

The exact incidence of ACPO is unknown. It occurs in about 1% of hospitalized patients and 0.046% of patients undergoing CABG surgery. The prevalence is higher in late middle age (around 60 years) and it is slightly more common in men [8].

The pathophysiology is not completely understood. The most widely accepted theory is due to an imbalance in the autonomic regulation of colonic motor function, which leads to excessive parasympathetic suppression or sympathetic stimulation or both. The parasympathetic nervous system increases contractility, whereas the sympathetic nerves decrease motility in the colon [6, 8].

Patients present initially with abdominal distension, which usually develops over 3–7 days. Other clinical features of ACPO include abdominal pain, nausea, and vomiting. About 40% of the patients will continue to have passage of flatus or stool. Physical examination confirms that a dilated, tympanic abdomen and bowel sounds are usually present but hypoactive [1, 9].

Plain abdominal X-rays demonstrate colonic dilatation with the right colon and cecum being the most dilated structures. The differential diagnosis should include mechanical obstruction, fecal impaction, volvulus, and toxic megacolon [10].

Diagnosis is suggested by the clinical presentation and abdominal radiography. The right colon and cecum show the most marked distension, with possible cutoffs at the splenic flexure or descending colon. Computed tomography is usually done to rule out obstructive lesions or possible bowel ischemia [5, 6].

If left untreated, Ogilvie's syndrome may lead to life-threatening complications such as bowel ischemia or spontaneous perforation. Patients with ischemic or perforated bowel usually have clinical findings of peritonitis and are likely to be febrile [6].

The mortality rate is about 14% in cases treated conservatively, 30% in patients undergoing surgical treatment, and up to 40%–50% when ischemia or perforation occurs [1, 11].

The risk of colonic perforation increases significantly when the cecal diameter exceeds 12 cm and when distention has been present for more than six days [8].

Conservative management should be the initial approach to treatment; supportive measures include keeping the patient NPO, insertion of a nasogastric tube, intravenous fluid administration, correction of electrolyte abnormalities, insertion of a rectal tube, and discontinuation of medications that have an effect on bowel motility such as opiates, anticholinergics, and calcium channel blockers [10].

Patients who fail supportive therapy are candidates for further intervention. Medical therapy with neostigmine, a reversible acetylcholinesterase inhibitor, is the initial therapy of choice [1]. Neostigmine indirectly stimulates muscarinic parasympathetic receptors, enhancing colonic motility. Administered intravenously, neostigmine has a rapid onset of action. A study by Ponec et al. showed the median response time with intravenous neostigmine to be 4 minutes. Using a dose of 2 mg by intravenous infusion over 3–5 min, a clinical response was observed in 91% of patients randomized to receive neostigmine [8].

Urgent endoscopic colonic decompression is the initial invasive procedure of choice and is indicated in patients who have contraindications to or fail pharmacological therapy with neostigmine. A successful colonoscopic decompression is determined by a reduction in radiographically measured cecal diameter. The recurrence rate of approximately 40 percent may be decreased by placement of a decompression tube at the time of the procedure.

Surgical intervention is reserved for patients who fail endoscopic and pharmacologic management and those with signs of bowel ischemia or perforation. The type of surgery performed depends on the status of the bowel. Without perforated or ischemic bowel, percutaneous cecostomy is the procedure of choice, with a high success rate and a relatively low morbidity. In the case of an ischemic or perforated bowel, segmental or subtotal colectomy is indicated, with either a colostomy formation or primary anastomosis [8, 10].

## 4. Conclusion

Ogilvie's syndrome can be a potentially fatal disorder. In hospitalized patients who develop constipation and abdominal distension after undergoing a procedure, Ogilvie's syndrome should be highly considered in the differential diagnosis. Initial management is conservative, followed by medical therapy with neostigmine in patients who fail to improve. Surgical intervention may be warranted in patients failing medical therapy or in complicated cases. To our knowledge, no association has ever been described between ACPO and cardioversion for atrial fibrillation. We hypothesize that Ogilvie's syndrome has possibly developed secondary to an imbalance in the autonomic regulation of colonic motor function induced by the electric current during cardioversion.
